# Dietary Supplement Interventions and Sleep Quality Improvement: A Systematic Review and Meta-Analysis

**DOI:** 10.3390/nu17243952

**Published:** 2025-12-17

**Authors:** Meijuan Mei, Qiya Zhou, Wenting Gu, Feifei Li, Ruili Yang, Hongtao Lei, Chunhong Liu

**Affiliations:** 1 College of Food Science, South China Agricultural University, Guangzhou 510642, China; su_su1434127940@163.com (M.M.); 15889974739@163.com (Q.Z.); gwtgz2020@126.com (W.G.); l18439692270@163.com (F.L.); rlyang77@163.com (R.Y.); hongtao@scau.edu.cn (H.L.); 2 Guangdong Provincial Key Laboratory of Food Quality and Safety, Guangzhou 510642, China

**Keywords:** dietary intervention, dietary nutrients, sleep disorder, sleep improvement, meta-analysis

## Abstract

Background/Objectives: Sleep health impacts numerous domains of human health, and sleep deprivation has emerged as a significant public health concern. Multiple types of dietary nutrient supplementation, dietary intake, and the use of nutritional supplements to enhance sleep quality are recognized as effective methods to improve sleep quality. Methods: We aim to systematically evaluate the efficacy of dietary supplement interventions in sleep quality improvement across populations with sleep disorders and healthy individuals. We conducted a comprehensive literature search across PubMed, Web of Science, CNKI, ScienceDirect, Wiley, and CVIP databases. Sleep evaluation metrics included the Pittsburgh Sleep Quality Index (PSQI), sleep efficiency (SE), sleep latency (SL), total sleep time (TST), wake after sleep onset (WASO), and number of awake after sleep onset (NASO). Meta-analysis procedures were executed in Review Manager 5.3 and Stata 17.0, with heterogeneity quantified via *I*^2^ statistics. Results: This study, in total, included 28 randomized controlled trials. This meta-analysis’s results suggest dietary interventions significantly improved sleep outcomes: reduced PSQI (MD: −0.70, 95% CI: −1.37 to −0.03, *p* < 0.05), increased SE (+2.58 min, 95% CI: 2.01–3.16, *p* < 0.00001), prolonged TST (SMD: +0.23, 95% CI: 0.04–0.43, *p* < 0.05), and shortened SL (SMD: −0.24, 95% CI: −0.37 to −0.10) and WASO (SMD: −0.30, 95% CI: −0.48 to −0.12) (both *p* < 0.001). NASO showed a marginal reduction (MD: −1.57, 95% CI: −3.16 to 0.02, *p* = 0.05). Conclusions: These findings suggest that tryptophan, vitamin D, omega-3, zinc, and antioxidants may enhance sleep quality by decreasing SL, and WASO increases SE and extends TST, respectively.

## 1. Introduction

Sleep disorders, conceptualized as conditions that disturb normal sleep patterns, encompass six major categories, including insomnia disorders, sleep-related breathing disorders, central disorders of hypersomnolence, circadian rhythm sleep–wake disorders, sleep-related movement disorders, and parasomnias [1]. These conditions can lead to clinical sleep disorders such as sleep apnea and hypersomnia. The significance of sleep extends far beyond mere rest; it is fundamental to neurological, psychological, and immune functioning [2]. An article in *The Lancet* highlights insufficient sleep as a growing public health crisis, demonstrating that sleep deprivation elevates the risk of chronic conditions, including neurodegenerative, cardiovascular, and metabolic diseases, as well as psychiatric illnesses [3].

This crisis is fueled by modern living. Compounding stressors across life domains, exacerbated by noise pollution and poor lighting conditions, collectively drive sustained deterioration in sleep quality [4,5,6,7]. Insomnia prevalence has exhibited a consistent upward trend over recent decades. Data from the 2024 White Paper on Sleep Health of Chinese Residents reveal that over 50% of the population experiences suboptimal sleep, characterized by symptoms such as nocturnal awakenings, early morning awakening, and difficulty falling asleep. As a critical public health challenge, sleep disorders erode global wellness and compound burdens on both healthcare systems and socioeconomic structures. This underscores the urgent need for comprehensive research to elucidate the mechanisms behind sleep issues, develop preventive protocols, and refine therapeutic strategies [8].

Contemporary treatment for sleep disorders ranges from pharmacological and cognitive–behavioral interventions to physical therapies, over-the-counter (OTC) products, and herbal or dietary supplements [9,10,11]. In clinical practice, pharmacotherapy is often the mainstay of treatment. This typically involves the administration of sleep-promoting medications, such as benzodiazepine receptor agonists or melatonin (MT), prior to bedtime [12]. However, this approach is associated with the risk of adverse drug reactions (e.g., agitation, amnesia, cognitive impairment, confusion, depression, and dementia) [13], dependence, and addiction, which pose significant threats to physical health. In addition, the research conducted by D. Liu and S. Kanji revealed that the pharmaceutical treatment group did not demonstrate a significant improvement in sleep quality when compared to the placebo control group [14,15]. Physical therapy demands professional devices, and the high cost of equipment and the requirement of operating technology limit the practical usability for most groups [16]. Cognitive behavioral therapy for insomnia (CBT-I) is an effective and safe intervention, as well as a first-line recommendation and primary non-pharmacological treatment for sleep disorders. However, its effectiveness is limited by its heavy reliance on patient engagement and adherence, which can be challenging to maintain [17,18,19]. With rising living standards, there is a growing interest in dietary nutrition due to its favorable safety profile. Consequently, the consumption of foods enriched with specific sleep-improving functional components is likely to become increasingly widespread among individuals with sleep disorders [20,21]. The objective of this research is to systematically synthesize evidence from randomized controlled trials (RCTs) on the effects of dietary interventions—specifically, the consumption versus non-consumption of nutrient-rich foods or supplements—on sleep quality. This study aims to appraise the efficacy and safety of these interventions and to provide guidance for developing effective functional foods and strategies to address poor sleep quality in patients with sleep disorders.

## 2. Methods

### 2.1. Protocol and Registration

The research was conducted according to the Cochrane handbook for systematic reviews of interventions progression [22,23], and follows the guidelines of the Preferred Reporting Items for Systematic Reviews and Meta-Analyses (PRISMA). This research has already been registered with PROSPERO (CRD420250656302; website: https://www.crd.york.ac.uk/PROSPERO/).

### 2.2. Search Strategy

The search strategy was designed to combine Medical Subject Headings (MeSH) terms with synonyms to ensure both accuracy and comprehensiveness. We searched six databases—PubMed, Web of Science, ScienceDirect, Wiley, CNKI, and CVIP—from their inception to 15 November 2024. The detailed search formulas are provided in Table S1.

### 2.3. Inclusion Criteria

We followed the PICOS criteria to select the literature and decide whether it could be included (Table 1). As an important part of study selection, the detailed criteria are as follows: (1) study object: An adult (≥19 years) who is healthy or suffering from sleep disorders related to diseases; (2) Intervention: The study primarily refers to food(s) or dietary nutrition intake; (3) Comparison: Comparing the effect of intervention between the dietary nutrition intake intervention group and placebo group. (4) Outcome index: Reporting the changes in sleep efficiency (SE), sleep latency (SL), total sleep time (TST), wake after sleep onset (WASO), number of awake after sleep onset (NASO), subjective sleep index, and the Pittsburgh Sleep Quality Index (PSQI) before and after intervention; and (5) study design: Randomized control trial.

### 2.4. Study Selection

Firstly, the EndNote X9 software was used to eliminate duplicate articles. Then, two researchers independently screened the titles and abstracts to dig out potentially relevant studies. Finally, they read the full texts to determine whether the article could be included for use in the ultimate analysis. When a disagreement occurred, two reviewers were requested to discuss how to solve the problem comprehensively, or a third expert was consulted, and the discussion would stop until an agreement was reached. If the following reasons occurred, the study would be excluded: (1) The theme and (or) content did not conform to the theme of this study; (2) the study was not an RCT; (3) study object has diseases or complications apart from sleep disorder-relevant diseases; (4) the study’s data was missing; (5) the outcome indicator was not in the evaluation index of our study; and (6) the study lacked a placebo group.

### 2.5. Data Extraction

The information collected from each included study included the following: (1) Major authors and the date the articles were published; (2) study object’s age (mean age), gender, country, and health condition; (3) sample size; (4) the process and duration of intervention; (5) study design; and (6) sleep quality outcome indicator. Results data was extracted either as change-from-baseline, baseline and post-intervention, or post-intervention means and standard deviations (SD). When the data report was incomplete, the researcher could calculate the mean (M) and standard deviation (SD) based on the sample size, median, range, and *p*-value of the study report according to the Cochrane handbook [24]. If the data was presented graphically and the author of the article cannot be contacted, GetData Graph Digitizer 2.26 software was used to extract the data to obtain the mean and standard deviation according to the Cochrane handbook.

### 2.6. Study Risk of Bias Assessment

Two researchers evaluated the risk of bias of the included studies independently according to the Cochrane bias risk assessment tool [22,25,26], and a third researcher conducted the ultimate assessment [24]. The Cochrane bias risk assessment tool mainly covers six aspects, including selection bias, performance bias, detection bias, attrition bias, reporting bias, and other biases [26]. Risk of bias is divided into three degrees: when a study is proven to lack randomization or has insufficient randomization and allocation of randomization, and there is non-double-blinding between subjects and/or study investigators and outcome assessments, it will be defined as high risk of bias. Conversely, risk of bias is considered low when there is sufficient randomization and allocation concealment, double-blindness between subjects and (or) research investigators, and outcome assessments [27].

### 2.7. GRADE Assessment

The quality assessment of evidence is necessary for a systematic review. A GRADE assessment was taken for indices of sleep quality outcome, with evidence rated as “high”, “moderate”, “low”, or “very low” according to the confidence as to whether the true effect lies close to the estimated effect [28]. There are six domains evaluated for each outcome: study design, risk of bias, inconsistency, indirectness, imprecision, and publication bias. Using the online tool GRADEpro (https://www.gradepro.org), two review authors independently performed the quality assessments. Any disagreements between them were resolved by consensus or with the arbitration of a third author.

### 2.8. Statistical Analysis

The mean and standard deviation of the results before and after the intervention were extracted from each study, and the changes between the experimental group and control group before and after the intervention were utilized to create a forest map to estimate general effects. If there is no pre-intervention or baseline data in a study and the original author cannot be contacted, the pre-intervention value is predicted by constructing a regression model [29,30]. Two experimental or control groups closely related to the research topic were merged according to the following formulas:

$$M_{pooled}\;=\;\frac{N_{1}M_{1}\;+\;N_{2}M_{2}}{N_{1}\;+\;N_{2}}$$

$${SD}_{pooled}=\sqrt{\frac{\left(N_{1}-1\right){\mathrm{SD}}_{1}^{2}+\left(N_{2}-1\right){\mathrm{SD}}_{2}^{2}+\frac{N_{1}N_{2}}{N_{1}+N_{2}}(M_{1}^{2}+M_{2}^{2}-2M_{1}M_{2})}{N_{1}+N_{2}-1}}$$

Among them, it is assumed that the sample size of the experimental group A is N_1_, the mean is M_1_, and the standard deviation is SD_1_; the sample size of the intervention group B is N_2,_ the mean is M_2_, and the standard deviation is SD_2_. If three or more experimental or control group data need to be combined, two intervention groups can be combined, in turn, according to the above formulas, and then the results are combined with the next group until the combination of all groups of data is completed [31]. MDs were calculated when the measurement tool was the same across all studies, and the SMDs were calculated when the measurement tools differed or when there were substantial numerical differences between studies (e.g., means or standard deviations differ by a factor of 10 or more), but the same behavioral outcome was assessed.

When analyzing the outcomes, it is necessary to combine 95% confidence interval (CI) (95% CI) value and *I*^2^ for analysis. When using *I*^2^ to evaluate the heterogeneity of the study, high heterogeneity is indicated by *I*^2^ over 50%, and a random effect model is chosen as the effect model; otherwise, the heterogeneity is low, and the fixed-effect model is selected [22,32,33]. Subgroup analysis can be used to assess the potential reasons for heterogeneity. But we required at least four studies in each subgroup, and the more studies in each subgroup, the more accurate the potential causes of heterogeneity [34,35,36]. Additionally, sensitivity analysis was performed on the included studies to evaluate the robustness and reliability of the results. The meta-analysis was conducted, and risk of bias assessment, forest plots, sensitivity analysis, and subgroup analysis were performed in the Review Manager software (V.5.4), and the funnel plots and meta-regression were performed in Stata software (V.17.0).

## 3. Results

### 3.1. Included Studies

**Figure 1:**
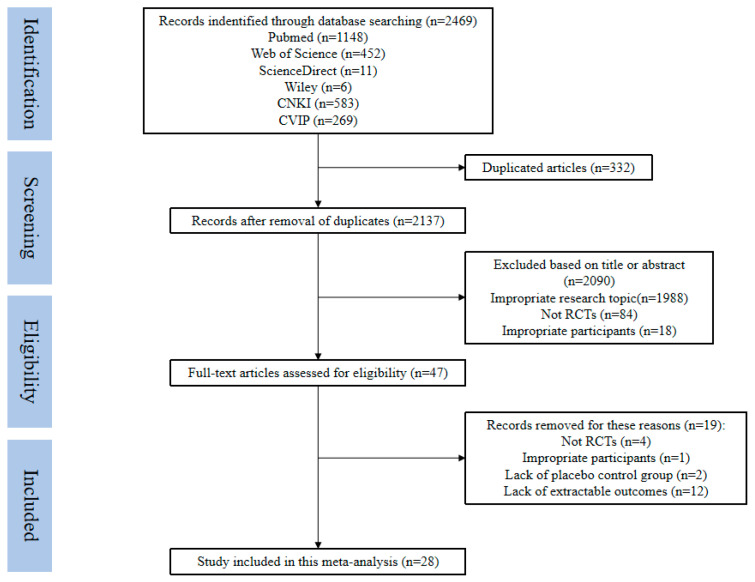
Flow diagram of study screening and selection process.

A sum of 2469 related records were obtained in the preliminary search. Of these records, 28 studies [37,38,39,40,41,42,43,44,45,46,47,48,49,50,51,52,53,54,55,56,57,58,59,60,61,62,63,64] were identified that met the inclusion criteria. Importing the literature into EndNote X9 software removed 332 duplicates, leaving 2137 records. A preliminary selection of titles and abstracts was conducted to screen out studies that met the following criteria: the theme did not align with the research topic, the content was not related to our study, the study design was not an RCT, and the subjects had inappropriate age or other diseases and complications. A total of 2090 records were found to meet the above exclusion criteria. The remaining 47 publications were then subjected to a thorough review, during which studies lacking complete data, placebo control groups, or appropriate assessment indicators were excluded. Following a thorough examination, 28 studies were deemed suitable for meta-analysis. We did not contact the corresponding authors to acquire research data because the 28 studies were enough to perform a meta-analysis. The screening process is shown in Figure 1.

### 3.2. Study Characteristics and Individual Outcomes

The summary of the general characteristics of the included studies is presented in Table S2. The publication dates of the studies ranged from 1991 to 2024. The sample sizes of the studies ranged from 7 to 50 subjects, with the majority of the subjects falling within the 20 to 65 age range. The subjects included in the studies were either healthy or suffered from sleep disorders, but did not suffer from other illnesses or complications, or they were recovering from an illness. The majority of studies included both male and female subjects, while three focused exclusively on males [42,46,52], two focused on females [51,56], and one did not report gender [41]. The duration of the interventions ranged from 1 to 26 weeks, with individual studies lasting less than one week [43,58]. The dietary interventions encompassed food or dietary supplements, categorized into amino acids and derivatives, vitamins, trace elements, unsaturated fatty acids, natural extracts, and proteins. The most commonly utilized indicators included PSQI, SE, TST, SL, WASO, and NASO. The assessment of sleep quality was predominantly conducted using a combination of indicators, with the majority of studies employing a minimum of two indicators, while individual studies evaluated with only one indicator [38,39,40,43,51,55].

### 3.3. Risk of Bias, Publication Bias, and Certainty Assessment

**Figure 2:**
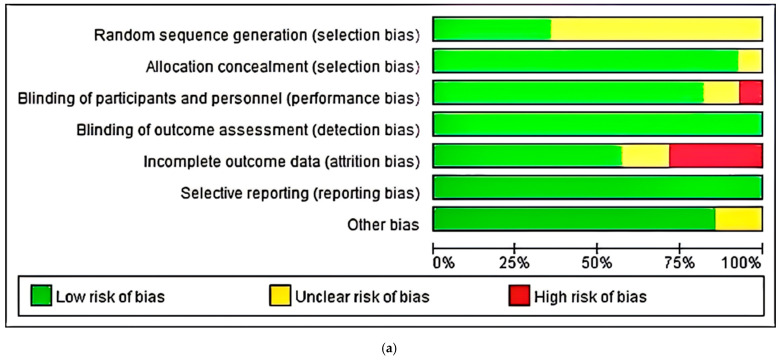

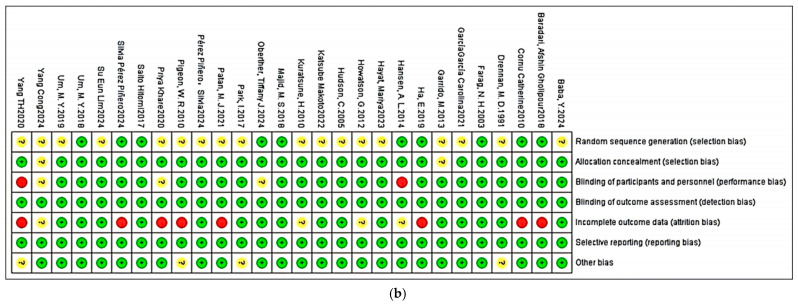
Risk of bias assessment for trials. (**a**) Result of risk of bias assessment for trials; (**b**) detailed risk assignment of the individual RCTs [37,38,39,40,41,42,43,44,45,46,47,48,49,50,51,52,53,54,55,56,57,58,59,60,61,62,63,64].

The risk of bias assessment of the 28 RCTs selected for this review is shown in Figure 2a, and a detailed risk assessment diagram is shown in Figure 2b. Figure 2 shows that most of the included studies were of high quality, and the high risk of bias in a few was mainly due to bias caused by attrition during the intervention. However, the risk of selection bias of the included studies in the description of randomized sequence generation remains unclear. Incomplete randomization or error randomization may influence the accuracy of meta-analysis, and it may cause high heterogeneity that influences the authority of study outcomes. Therefore, the sensitivity statistics, meta-regression, and subgroup analysis were taken to assess the robustness of results and the relationship between risk of bias and effect estimation.

**Figure 3:**
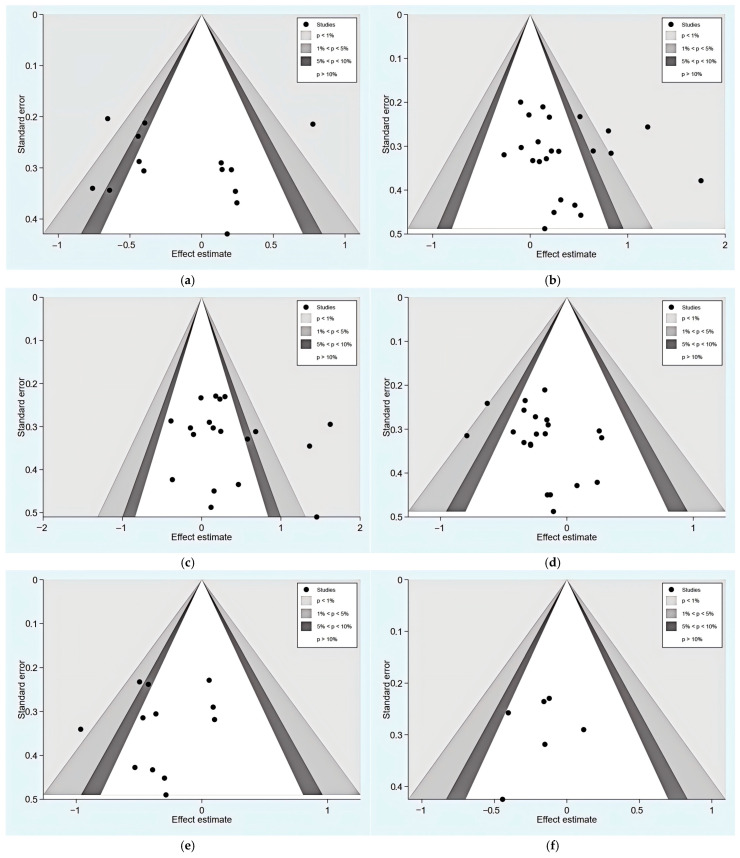
Contour-enhanced funnel plots of studies analyzing the effect of dietary intake. (**a**) Contour-enhanced funnel plots of PSQI under the random-effects model; (**b**) contour-enhanced funnel plots of SE under the fixed-effects model; (**c**) contour-enhanced funnel plots of TST under the random-effects model; (**d**) contour-enhanced funnel plots of SL under the fixed-effects model; (**e**) contour-enhanced funnel plots of time to WASO under the fixed-effects model; (**f**) contour-enhanced funnel plots of NASO under the fixed-effects model.

Funnel plots serve as a visualization method for examining the distribution of study outcomes and exploring potential publication bias. While symmetrical plots typically indicate minimal bias, observed asymmetry in these graphical representations may reflect certain limitations in the evidence synthesis process. This pattern may be partially explained by methodological characteristics of the included research, such as studies with relatively smaller sample sizes or less rigorous randomization procedures, potentially contributing to effect size variations. In the current analysis, both fixed-effect and random-effects models were applied to evaluate publication bias across multiple outcome measures using contour-enhanced funnel plots and Egger test assessments. The graphical outputs (Figure 3) reveal varying degrees of asymmetry, which may be associated with either potential publication bias or underlying heterogeneity among studies. Contour plots improved interpretation by demarcating statistical significance thresholds (e.g., *p* < 0.01, *p* < 0.05), helping distinguish publication bias from artifacts such as heterogeneous outcome measures or small-sample bias [65]. The Egger test, a statistical method assessing funnel plot asymmetry in meta-analysis, utilizes a weighted regression to examine the association between effect sizes and their standard errors, with a *p*-value (<0.05) indicating potential publication bias [66,67]. However, this test has limited statistical power in studies where fewer than 10 studies are analyzed and cannot distinguish between publication bias and heterogeneity arising from methodological differences or clinical variability. We, therefore, conducted Egger tests to evaluate whether the observed asymmetry primarily reflected publication bias. The resulting *p*-values for PSQI (0.765), SE (0.365), TST (0.408), SL (0.151), WASO (0.545), and NASO (0.666) all exceeded 0.05, indicating that publication bias was unlikely to be the primary source of funnel plot asymmetry. To better understand these patterns, subsequent sensitivity analyses were conducted to examine how specific study characteristics influence the overall results. Such explorations help to clarify whether the observed asymmetries substantially affect the robustness of our conclusions.

### 3.4. Meta-Analysis Results

In this study, sleep quality was assessed using the PSQI, a self-reported sleep assessment index, and the SE, TST, SL, WASO, and NASO, which are multiple outcome indicators of objective sleep assessment recorded by instruments. A meta-analysis was conducted to evaluate the effects of dietary supplementation on the PSQI, SE, TST, SL, WASO, and NASO. This meta-analysis employed a combination of random-effects and fixed-effects models to analyze the data. The datasets of the experimental and control groups included in the meta-analysis were the post-intervention versus pre-intervention change values for each group. The forest plots were then utilized to visually represent the effect sizes of each study and the combined effect sizes. For indicators exhibiting substantial heterogeneity, meta-analysis, subgroup analyses, and sensitivity analyses were employed to identify the underlying causes of heterogeneity.

#### 3.4.1. Pittsburgh Sleep Quality Index

**Figure 4:**
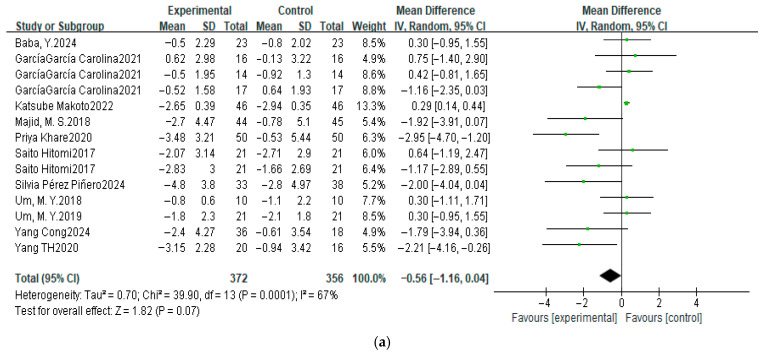

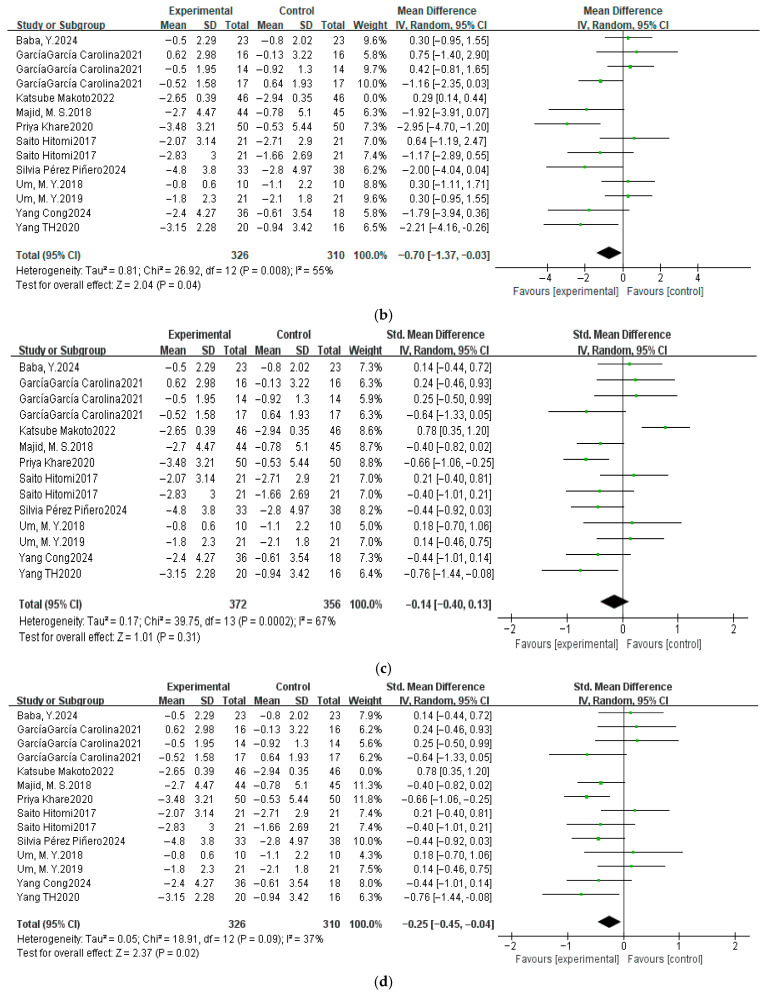
Random-effects model meta-analysis in PSQI (scores) after dietary supplementation. Analysis using MD (**a**) before sensitivity analysis, and (**b**) after sensitivity analysis. Analysis using SMD (**c**) before sensitivity analysis, and (**d**) after sensitivity analysis [37,39,41,48,51,54,55,56,62,63,64].

A meta-analysis was conducted on 14 datasets from 11 studies to assess the effect of dietary intervention on PSQI scores. As shown in the forest plot (Figure 4), significant heterogeneity was observed (χ^2^ = 39.90, *p* < 0.01, *I*^2^ = 67%), indicating substantial variability between studies. Consequently, a random-effects model was applied.

The pooled mean difference (MD) was −0.56 (95% CI: −1.16, 0.04) and the overall effect was 1.82 (*p* = 0.07), which did not reach statistical significance. Similarly, the pooled standardized mean difference (SMD) was −0.14 (95% CI: −0.14, 0.13) and the overall effect was 1.01 (*p* = 0.31), which was also non-significant.

Although the point estimates for both MD and SMD were negative—suggesting a potential trend favoring the experimental group—the 95% CI crossed zero and the *p*-values were greater than 0.05. Therefore, this meta-analysis did not find sufficient evidence to conclude that dietary supplementation significantly reduces PSQI scores or improves sleep quality.

#### 3.4.2. Sleep Efficiency

**Figure 5:**
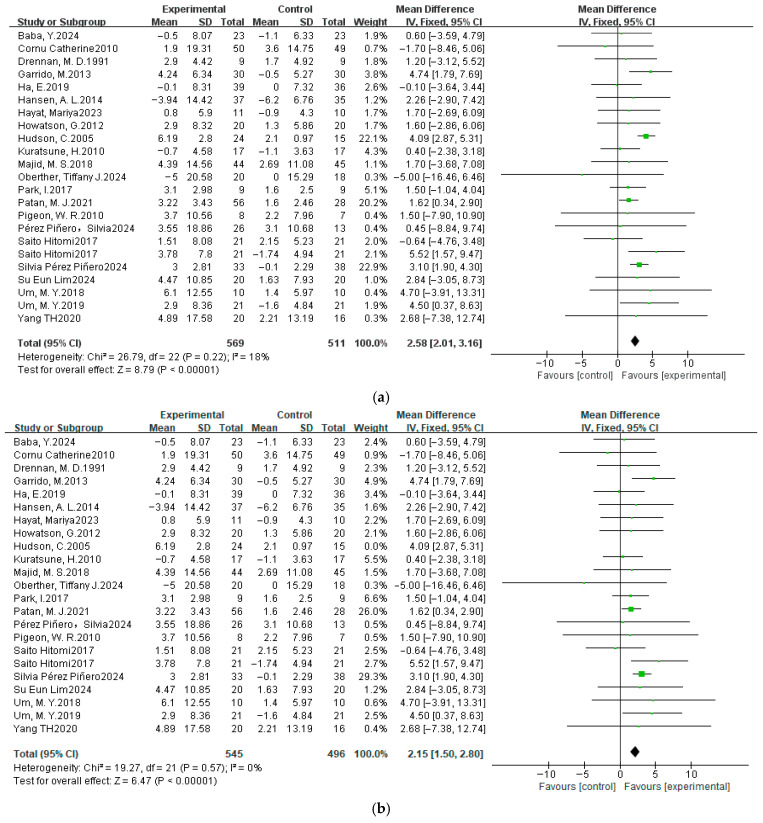
Fixed-effects model meta-analysis comparing MD in SE (%) after dietary supplementation. (**a**) Before sensitivity analysis, and (**b**) after sensitivity analysis [37,40,42,44,45,46,47,48,49,50,52,53,54,56,57,58,59,60,61,62,63,64].

A total of 24 datasets from 22 studies were subjected to meta-analysis. As illustrated in Figure 5, the forest plot of the dietary intervention on SE indicators showed the magnitude of heterogeneity with χ^2^ = 26.79 and *p* > 0.05. The *I*^2^ value was 18%, indicating low heterogeneity among the studies, which were analyzed using a fixed-effects model. The pooled MD was 2.58 (95% CI: 2.01, 3.16), with a significant overall effect (Z = 8.79, *p* < 0.05). The results in the Figure also showed that the pooled effect size favored the experimental group, which indicates that dietary supplementation may increase SE and effectively improve sleep quality.

#### 3.4.3. Total Sleep Time

A meta-analysis was conducted on 19 datasets from 18 studies to evaluate the effect of dietary interventions on total sleep time (TST). Given the substantial variations in standard deviations across studies, standardized mean differences (SMDs) were selected as the primary effect size.

**Figure 6:**
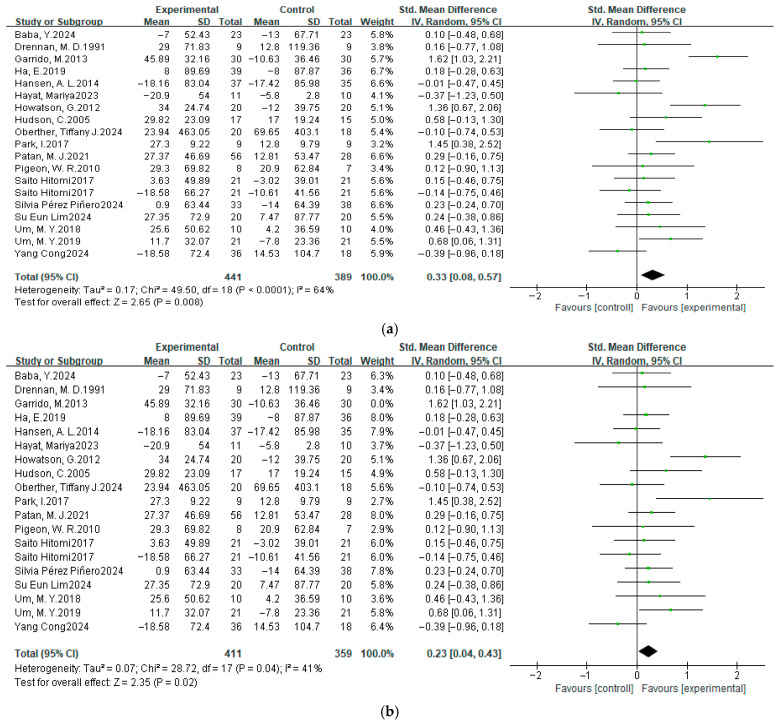

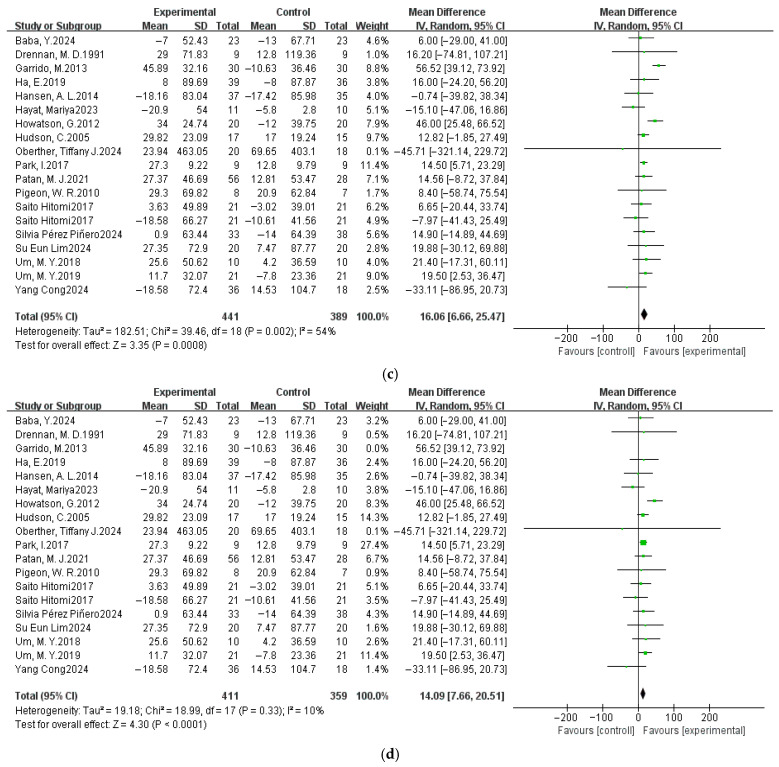
Random-effects model meta-analysis in TST (min) after dietary supplementation. Analysis using SMD (**a**) before sensitivity analysis, and (**b**) after sensitivity analysis. Analysis using MD (**c**) before sensitivity analysis, and (**d**) after sensitivity analysis [37,41,42,44,45,46,47,48,49,50,53,57,58,59,60,62,63,64].

Analysis using SMD: The forest plot (Figure 6a,b) revealed significant heterogeneity (χ^2^ = 49.50, *p* < 0.05, and *I*^2^ = 64%). A random-effects model was, therefore, applied. The pooled SMD was 0.33 (95% CI: 0.08, 0.57), indicating a statistically significant overall effect favoring the experimental group (Z = 2.65, *p* < 0.05).

Analysis using MD for comparison (Figure 6c,d): For comparison, we also calculated the pooled MD. The heterogeneity was similar (χ^2^ = 39.46, *p* < 0.01, and *I*^2^ = 54%). Using a random-effects model, the pooled MD was 16.06 (95% CI: 6.66, 25.47), which also showed a statistically significant effect (Z = 3.35, *p* < 0.01).

Both analyses yielded consistent results: The pooled effect sizes significantly favored the experimental group. This suggests that dietary supplementation may increase total sleep time, potentially improving sleep quality.

#### 3.4.4. Sleep Latency

**Figure 7:**
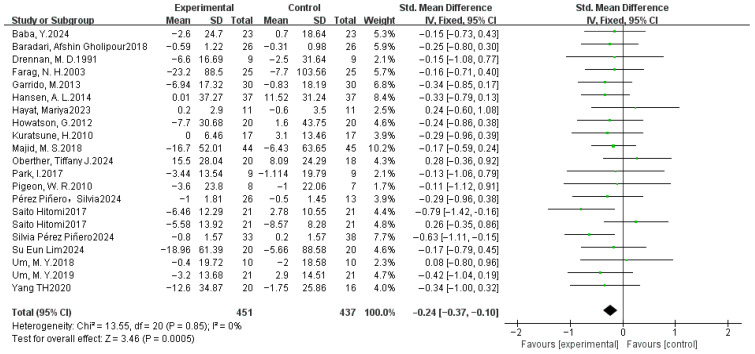
Fixed-effects model meta-analysis comparing SMD in SL (min) after dietary supplementation [37,38,42,43,44,45,46,47,48,49,50,52,53,54,55,56,57,58,60,61,62,63,64].

A meta-analysis was conducted on 21 datasets from 20 studies. SMDs were selected as effect sizes due to the substantial variation in standard deviations observed among the included studies. The forest plot of the dietary intervention on SL indicators (Figure 7) revealed low heterogeneity (χ^2^ =13.55, *p* > 0.05, *I*^2^ = 0%), justifying the use of a fixed-effects model. The pooled SMD between the experimental and control groups was −0.24 (95% CI: −0.37, −0.10), with a significant overall effect (Z = 3.46, *p* < 0.05). The results indicated that the pooled effect size favored the experimental group, suggesting that dietary supplementation may shorten sleep latency and improve sleep quality.

#### 3.4.5. Wake After Sleep Onset

**Figure 8:**
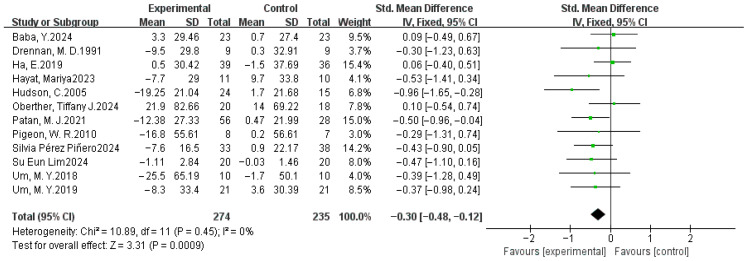
Fixed-effects model meta-analysis comparing SMD in WASO (min) after dietary supplementation [37,42,45,47,50,53,57,59,60,62,63,64].

A meta-analysis was conducted on a dataset comprising 12 studies. Standardized means were selected as effect sizes due to the substantial variation in standard deviations observed among the included studies. The forest plot of the dietary intervention on WASO indicators (Figure 8) revealed a low heterogeneity (χ^2^ = 10.89, *p* > 0.05, *I*^2^ = 0%), justifying the use of a fixed-effects model. The pooled SMD between the experimental and control groups was −0.30 (95% CI: −0.48, −0.12), with a significant overall effect (Z = 3.31, *p* < 0.05). The results in the Figure also show that the pooled effect size favored the experimental group, which indicates that dietary supplementation may shorten the time of wakefulness after falling asleep and can significantly improve the quality of sleep.

#### 3.4.6. Number of Awake After Sleep Onset

**Figure 9:**
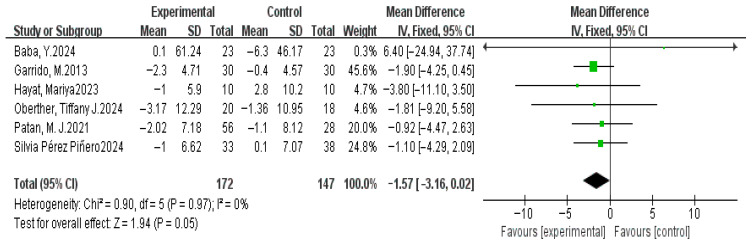
Fixed-effects model meta-analysis comparing MD in NASO (times) after dietary supplementation [37,44,47,57,59,62].

A meta-analysis was performed on six studies examining the effect of dietary interventions on the number of awake after sleep onset (NASO). As shown in the forest plot (Figure 9), the heterogeneity test revealed no significant variation across studies (χ^2^ = 0.90, *p* = 0.97, and *I*^2^ = 0%), supporting the use of a fixed-effect model. The pooled MD between the experimental and control groups was −1.57 (95% CI: −3.16, 0.02), with a marginally non-significant overall effect (*Z* = 1.94, *p* = 0.05). The confidence interval overlapped with the null line (effect size = 0), indicating that dietary interventions did not significantly reduce NASO.

### 3.5. Sensitivity Analysis

We performed sensitivity analyses for each meta-analysis to identify individual studies that may have influenced the between-study ratio. For the PSQI, when analyzed using MD, the heterogeneity *I*^2^ decreased from 67% to 55% (both *p* < 0.05) after the removal of one study [55], and the overall effect size became significant from Z = 1.82 (*p* = 0.07) to Z = 2.04 (*p* = 0.04). However, *I*^2^ decreased from 67% (*p* < 0.05) to 37% (*p* > 0.05) after the removal of the same study when using SMD, and the overall effect size became significant, with the value from Z = 1.01 (*p* = 0.31) to Z = 2.37 (*p* = 0.02, Figure 4). Screening other studies, the heterogeneity *I*^2^ did not change significantly. For the SE, after removing the Hudson et al. study [50], the heterogeneity χ^2^ = 26.79, *p* = 0.22, and *I*^2^ = 18% changed to χ^2^ = 19.27, *p* = 0.57, and *I*^2^ = 0%. The combined effect size changed from Z = 8.79 (*p* < 0.00001) to Z = 6.47 (*p* < 0.00001), and no significant change was observed when all other studies were excluded (Figure 5). For the TST, heterogeneity shifted from Tau^2^ = 0.17, χ^2^ = 49.50, *p* < 0.0001, and *I*^2^ = 64%, following the exclusion of Garrido et al. [44], to Tau^2^ = 0.07, χ^2^ = 28.72, *p* = 0.04, and *I*^2^ = 41%, and the overall effect size Z = 2.65 (*p* = 0.008) to Z = 2.35 (*p* = 0.02) when analyzed using SMD. In comparison, *I*^2^ decreased from 54% (*p* < 0.05) to 10% (*p* > 0.05) after the removal of the same study when using MD, and the overall effect varied from Z = 3.35 (*p* = 0.0008) to Z = 4.30 (*p* < 0.0001, Figure 6). The individual exclusion of the remaining studies did not result in a substantial alteration in the overall effect values or statistical significance. For SL, WASO, and NASO, the exclusion of any individual item did not yield a significant alteration in the composite effect values or statistical significance.

### 3.6. Meta-Regression

Meta-regression is a statistical technique used to investigate potential sources of heterogeneity across studies. Heterogeneity denotes the variability or inconsistency in results among different studies. By examining the association between study characteristics (e.g., population demographics, intervention details) and effect sizes, meta-regression helps identify which factors may explain such variability. If the *p*-value was less than 0.05, this may mean that the factor may be a potential source of heterogeneity.

In order to detect the potential factors for the high heterogeneity (PSQI, SE, and TST), meta-regression was performed in Stata software. The results of the meta-regression are shown below. It can be seen from the results of the meta-regression that the *p*-values of health condition, country, intervention time, and intervention type were 0.450, 0.015, 0.004, and 0.002, respectively. The results suggested that high heterogeneity of PSQI may probably come from the population’s country, intervention time, and intervention type (Table 2). As for SE, the *p*-value of intervention type was less than 0.05, suggesting SE’s heterogeneity may relate to intervention type (Table 3). And regarding TST, the *p*-value of country and intervention time were less than 0.05, and this may result in high heterogeneity (Table 4). And we further performed subgroup analysis on factors with *p*-values less than 0.05.

### 3.7. Subgroup Analysis

**Figure 10:**
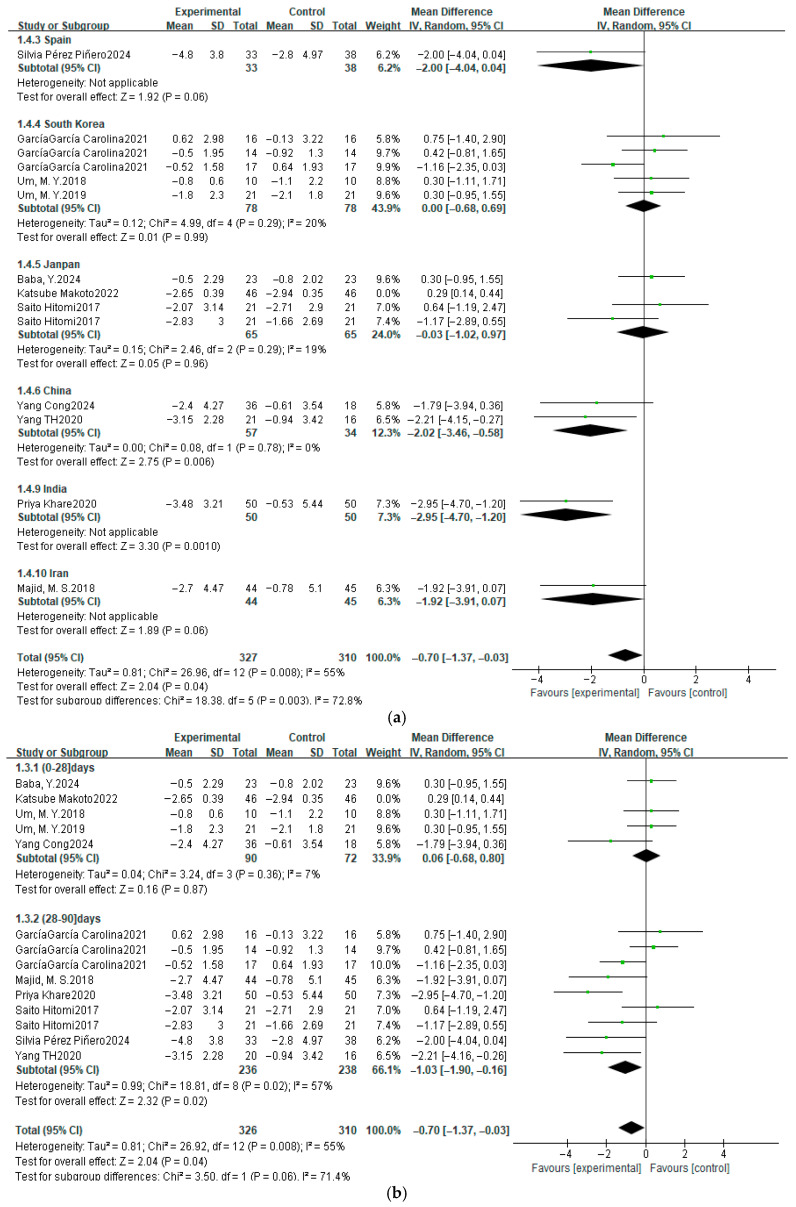

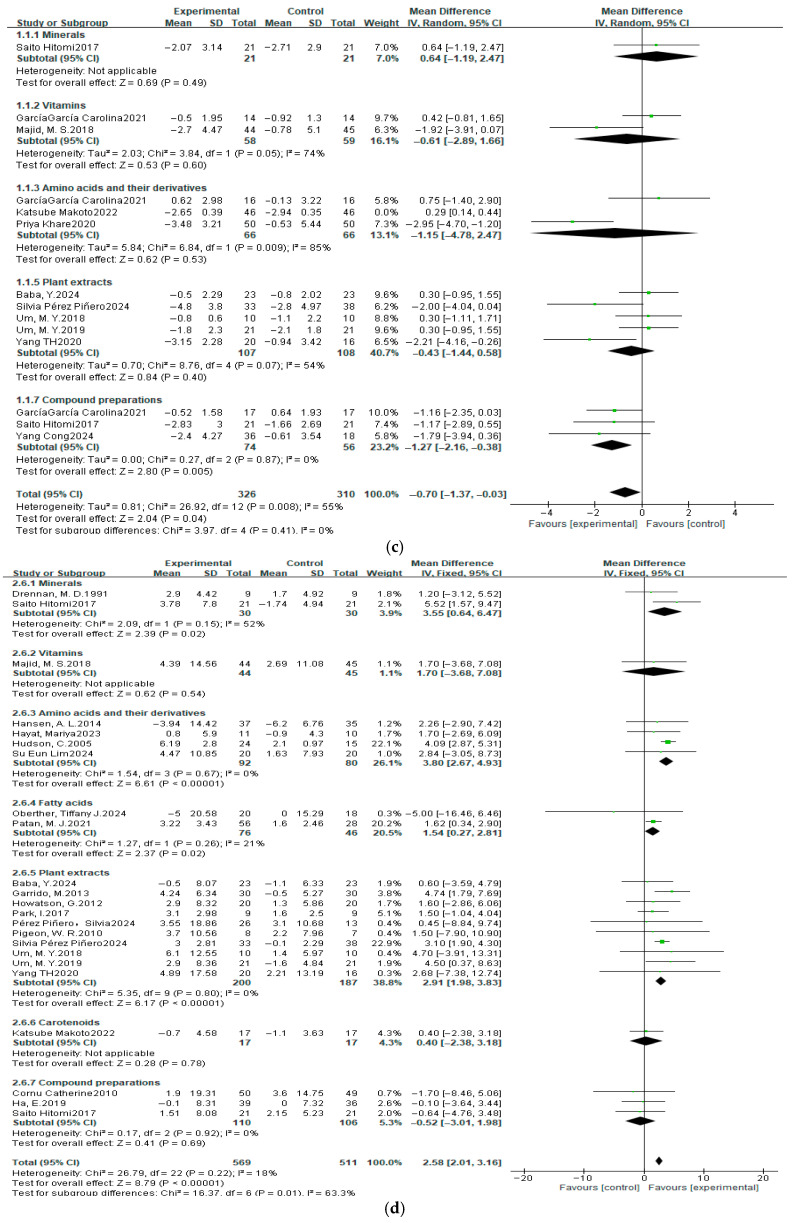

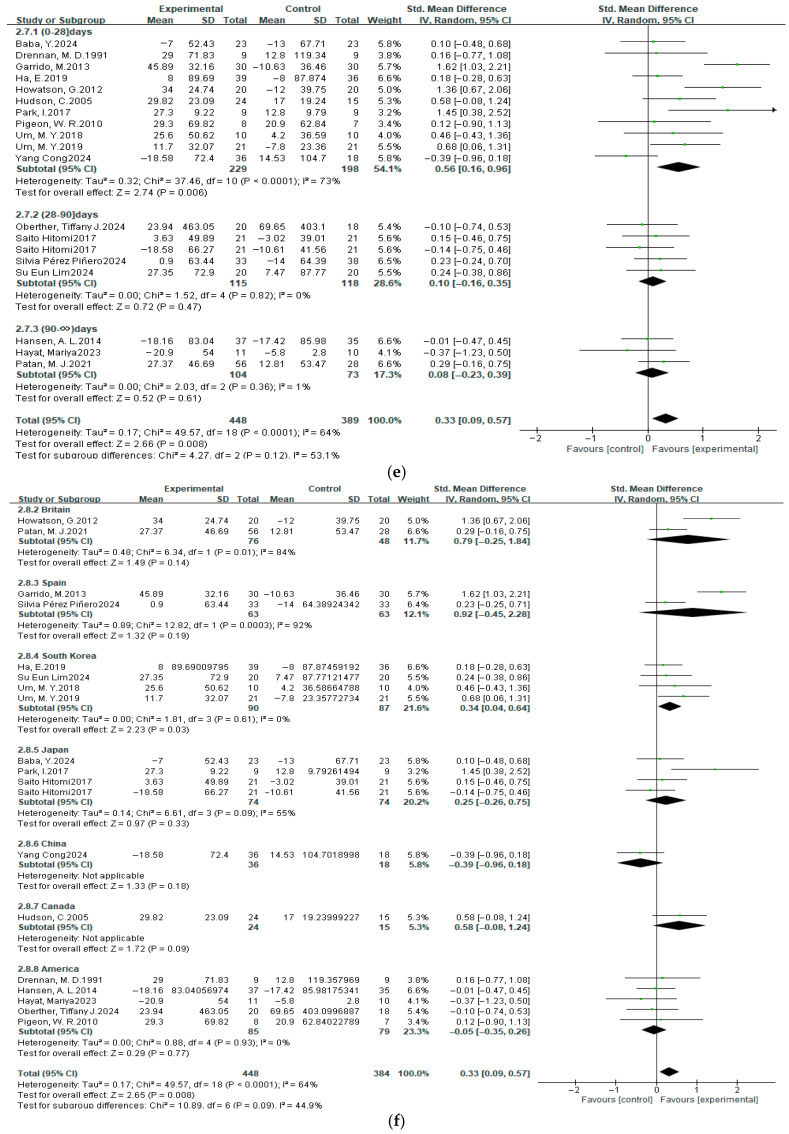
The results of subgroup analysis for PSQI, SE, and TST. (**a**) PSQI: the relationship between country attribution classification and heterogeneity; (**b**) PSQI: the relationship between intervention time and heterogeneity; (**c**) PSQI: the relationship between intervention type and heterogeneity; (**d**) SE: the relationship between intervention type and heterogeneity; (**e**) TST: the relationship between country attribution classification and heterogeneity; (**f**) the relationship between intervention time and heterogeneity [37,39,40,42,44,45,46,47,48,49,50,51,52,53,54,56,57,58,59,60,61,62,63,64].

Subgroup analysis was performed according to the study object’s country attribution classification, intervention time, and intervention types, and the outcomes were as follows (Figure 10).

According to subgroup analysis results for PSQI, we can infer that different intervention types are the major sources causing high heterogeneity (Figure 10a–c). The classification of amino acids and their derivatives varied greatly within the group (*I*^2^ = 85%). For SE, classification of minerals and amino acids and their derivatives have high heterogeneity within groups, and their *I*^2^ are 52% and 21%, respectively (Figure 10d). For TST, the intervention time less than 28 days’ subgroup has high heterogeneity (Figure 10e). And if the study object’s nationality is relevant to performing subgroup analysis, Britain, Spain and Japan are high heterogeneity within the subgroup (Figure 10f). The limitation study quantity in each subgroup may influence the accuracy of heterogeneity source inference. More importantly, the limited quantity of studies and the results of high heterogeneity may limit the promotion of research conclusions.

### 3.8. GRADE Assessment

The result of the GRADE assessment is shown in Table S3. According to the results, although all included studies were randomized controlled trials, none of the outcomes were rated as having ‘high’ certainty of evidence. And all outcomes were downgraded due to risk of bias, specifically attrition bias and/or performance bias. The evidence for SE, SL, TST, and WASO was graded as moderate. In contrast, PSQI and NASO were assigned a low grade because their 95% confidence intervals crossed the null line, prompting a one-level downgrade for imprecision. The conclusions of SE, SL, TST, and WASO were relatively certain: intervention may promote SE, prolong TST, shorten SL, and decrease WASO to improve sleep disorders. The conclusions of PSQI and NASO were uncertain, and this indicated that the intervention had the potential to slightly improve PSQI scores and reduce NASO; however, the estimate was imprecise and compatible with a negligible effect.

## 4. Discussion

This meta-analysis aimed to evaluate the efficacy of dietary interventions in improving sleep quality. Based on the predefined search strategy, 2469 records were initially identified. After applying the PICOS criteria for screening, 28 studies were ultimately included in this meta-analysis. The interventions encompassed foods or dietary supplements containing ingredients such as amino acids (and their derivatives), vitamins, trace elements, unsaturated fatty acids, natural extracts, and proteins.

We first evaluated the risk and publication bias of the included literature. Our results show that there are a few studies with no high risk, there is no large publication bias, and the quality of the included literature is high. High-risk studies are mainly due to the existence of incomplete randomization and error randomization, which also affects publication bias. The publication bias can be attributed to various factors, including the publication of substantial studies in languages other than English or in journals that do not adhere to open-source standards and are not indexed in the primary databases searched [68].

Our further meta-analysis revealed considerable heterogeneity in the pooled estimates for PSQI, SE, and TST, whereas SL, WASO, and NASO showed low heterogeneity. The participants, interventions, outcomes, research design, or intervention effects may result in high heterogeneity. To address this, we employed a random-effects model and performed sensitivity analyses, using either the MD or SMD as the effect size metric as appropriate. For SE, heterogeneity was substantially reduced following sensitivity analysis. For PSQI, heterogeneity showed no significant change under the MD model but was markedly reduced under the SMD model. For TST, high heterogeneity persisted in the SMD model before and after sensitivity analysis. In contrast, the MD model showed a significant reduction in heterogeneity after the same analysis. These results indicate that the choice of an appropriate effect size metric (MD vs. SMD) can critically influence the observed level of heterogeneity. The SMD is the appropriate effect size when the included studies measure the same continuous outcome using different scales or when their standard deviations differ substantially [69]. The standardization process converts the mean difference into a dimensionless metric, allowing for synthesis across these varied measurements. Conversely, for studies reporting the same continuous outcome on an identical scale, the MD is the preferred measure. SMD should be selected with caution in such cases, as it can perform less optimally than MD, potentially introducing greater bias, larger mean squared error, and lower confidence interval coverage [70]. Therefore, for PSQI, we selected the SMD because the studies used different scales, whereas for TST, we used the MD because all studies reported results on the same scale (minutes). The overall effects significantly favored the intervention group for PSQI (SMD model), SE, TST (MD model), SL, and WASO, except for NASO. The real-world effects of dietary interventions are often inconsistent due to specific components (e.g., the caffeine antagonism in matcha) and varying population characteristics. This heterogeneity can drive the pooled result in meta-analysis toward the null. Furthermore, if unpublished studies with negative results are included, the overall effect size may be attenuated further or shift closer to zero. Potential explanations for this finding include the limited quantity of included studies, high variability in outcome measurements (evidenced by inconsistent standard deviations across datasets), and possible publication bias, all of which may obscure the detection of a true intervention effect. Another probable reason is that, in addition to L-theanine, which has a positive effect on improving sleep, the matcha powder in the first study also contains caffeine, a substance that has a negative effect on sleep quality. Caffeine can block adenosine receptors that disrupt the sleep–wake cycle, which may increase the NASO. The dual side effect may be a key reason that the overall effect of NASO was close to the null line.

Finally, to investigate the sources of heterogeneity for PSQI, SE, and TST, we performed meta-regression and subgroup analyses. The meta-regression results indicated that the high heterogeneity might be attributable to differences in the participants’ country of origin (which may encompass variations in body composition), intervention duration, and the bioavailability of the supplement. All of these would play a critical role in a great intervention effect. Our subgroup analyses suggested that the specific classification of amino acids and their derivatives, as well as the nationality/country of participants, were potential major sources of the high heterogeneity observed. However, the limited number of studies within subgroups warrants caution in interpreting these findings.

This systematic review and meta-analysis suggested that dietary interventions may improve a set of sleep measures in patients with sleep disorders when compared to placebo. Based on the GRADE framework, we had moderate confidence in improvements for SE, SL, TST, and WASO. In contrast, the current evidence provides only low certainty for effects on NASO and patient self-reported PSQI scores, leaving their true effects unclear. Consistent with other studies in this field, the evidence certainty for PSQI and NASO outcomes was downgraded due to several limitations [71,72]. Attrition and performance bias were present across studies. Furthermore, substantial heterogeneity and inconsistency arose from large variations in effect sizes between trials. Finally, the overall imprecision—driven by small sample sizes and a confidence interval for the pooled effect that crossed the line of no effect—led to downgrading the evidence certainty. This judgment is reinforced by the sensitivity analysis: while the removal of certain outliers sometimes yielded a statistically significant result for PSQI, the effect estimate remained unstable and close to zero, underscoring the fragility of the conclusion. Similarly, for NASO, the point estimate suggests a reduction, but the upper limit of its confidence interval nearly touches the line of no effect, indicating that any benefit is not reliably established.

The pooled findings suggest that specific nutritional supplements, including proteins, amino acids or their derivatives, melatonin (MT), vitamins, unsaturated fatty acids, and antioxidant compounds, may enhance both subjective and objective sleep quality. These supplements were associated with reduced PSQI scores, shorter SL, fewer WASO and NASO, and increased SL and TST. However, heterogeneity in data reporting limited conclusive comparisons across supplement subtypes, as insufficient data were available for certain categories.

To place the findings within a robust scientific context, the biological plausibility of the results was examined through a discussion of potential mechanisms. These included the modulation of neurotransmitter synthesis (e.g., serotonin derived from tryptophan) and antioxidant-mediated circadian regulation. Studies provided sufficient data to draw definitive conclusions regarding the effects of each supplement type on sleep quality. However, a detailed discussion has been provided to explore the potential mechanisms by which these supplements might enhance sleep quality.

Proteins, amino acids, and their derivatives are indispensable for maintaining optimal human health. Notably, some protein sequences characterized by specific biological features are found to produce bioactive compounds (e.g., GABA, serotonin, and melatonin) upon gastrointestinal digestion, which have been consistently proven to promote sleep quality via regulating neurophysiological processes [73]. Substantial evidence from interventional studies confirms the sleep-enhancing potential of particular amino acids and their structural analogs, with notable examples including theanine, GABA, and tryptophan. Experimental investigations by T.P.R et al. [74] elucidated that L-theanine, an ethylamide derivative of glutamic acid predominantly present in tea, was a safe, natural sleep aid that improved the quality of sleep in all age groups. This amphipathic compound demonstrated dose-dependent sedative effects through dual mechanisms: competitive antagonism of glutamatergic neurotransmission via structural mimicry within the nervous system [75], coupled with selective enhancement of α-wave activity in the prefrontal cortex. Alpha-brain waves have been identified as a marker of relaxation. Administration of the amino acid L-theanine has been shown to induce relaxation by stimulating the production of alpha-brain waves [76,77]. Importantly, clinical trials confirmed that oral administration of L-theanine did not lead to dependence or other side effects. GABA possesses sleep-enhancing properties that are, in certain aspects, comparable to those of L-theanine. As the primary inhibitory neurotransmitter in the central nervous system, GABA is a key regulator of sleep. Its mechanism involves binding to and activating GABA_A_ receptors. This activation triggers the influx of chloride ions into neurons, leading to membrane hyperpolarization. The resulting suppression of neuronal excitability promotes nervous system relaxation, reduces sleep onset latency, and facilitates non-rapid eye movement (NREM) sleep [78]. These synergistic neurophysiological effects collectively improve sleep metrics. The investigation by Clarinda N. Sutanto et al. [27] revealed that supplementation with a specific amount of tryptophan significantly reduced WASO (−81.03 min/g; −1.08 (95% CI: −1.89, −0.28), *p* < 0.05), findings that align with our experimental observations. Bravo’s team [79] found that dietary intervention with tryptophan-rich grains exerted a positive effect on the sleep–wake cycle, demonstrating statistically significant improvements in sleep quality indices in elderly populations with chronic insomnia disorder. Biochemical studies identified tryptophan as a circadian regulator that modulates and maintains circadian rhythms [80]. Tryptophan is primarily metabolized in the body to produce MT, which plays a key role in improving sleep [81,82]. Melatonin exerts its sleep-improving effects through dual chronobiological mechanisms [83,84]. This dual action manifests clinically as 30.09 min reduction in sleep latency (*p* < 0.001) and 53.59 min extension of total sleep time (*p* < 0.01) in the comorbid insomnia population [85]. The study by Eduardo Ferracioli-Oda et al. yielded similar results, and overall sleep quality was significantly improved (Δ = 0.22, Z = 4.52, *p* < 0.001) [86]. Consequently, strategic dietary intake of tryptophan-rich foods such as milk, eggs, cheese, poultry, fish, etc., will enhance serum tryptophan availability, thereby potentiating melatonin biosynthesis and subsequent sleep optimization.

Accumulating clinical evidence substantiates the sleep-enhancing potential of vitamins, especially B vitamins and vitamin D, in improving sleep quality in populations with sleep disorders. As an essential cofactor for aromatic L-amino acid decarboxylase, vitamin B6 plays a role in improving sleep mainly by regulating the production of melatonin. Vitamin D exerts its somnotrophic effects through genomic regulation of tryptophan hydroxylase 2 (TPH2) expression [87,88]. Vitamin D deficiency slows the rate of slow-wave activity during restorative sleep, which can disrupt sleep homeostasis and circadian rhythm signaling [89]. In short, these micronutrients collectively mediate sleep optimization through two principal mechanisms: enzymatic facilitation of melatonin biosynthesis and epigenetic regulation of circadian clock gene expression.

The effects of unsaturated fatty acids (UFAs) on sleep quality are mediated through three primary mechanisms. Unsaturated fatty acids, such as the *n*-3 family of polyunsaturated fatty acids (PUFAs), may influence changes in circadian rhythms and regulate the sleep–wake cycle signal by modulating pineal gland function and affecting melatonin secretion. This biological pathway was substantiated by clinical research from M.J. Patan et al. [59], whose findings indicated that *n*-3 PUFA supplementation enhances sleep efficiency and reduces sleep latency through dual mechanisms: modulation of serotonin-MT biosynthetic pathways and optimization of sleep architecture parameters. These neurochemical and structural adaptations collectively contribute to improved sleep quality metrics [60,90].

Trace elements are indispensable for maintaining physiological homeostasis, with deficiencies potentially triggering systemic dysregulation. As the second most abundant trace metal element in the human body, zinc plays an important role in the sleep process. Current research consensus identifies zinc’s sleep-enhancing properties as primarily mediated through glutamatergic receptor interactions, which facilitate glutamate and melatonin release to improve sleep quality [91]. Studies have shown that magnesium also plays an important role in sleep regulation. It participates in the production and release of sleep-related neurotransmitters such as GABA and MT, while also regulating the activity of the hypothalamic–pituitary–adrenal axis to influence sleep [92,93,94]. Additionally, potassium ion channels are crucial for regulating neuronal excitability and activity. Research by Okamoto et al. demonstrated that a potassium-rich diet positively impacts sleep quality by promoting muscle relaxation, enhancing neurotransmitter transmission, and regulating slow-wave activity during sleep [95]. Meanwhile, leaky potassium channels contribute to maintaining normal sleep duration, as their dysfunction has been shown to reduce total sleep time [96]. Given these mechanisms, increased consumption of magnesium- and potassium-rich foods, including seeds and legumes, whole grains, nuts, and dark green vegetables, may effectively support sleep quality improvement [97].

Oxidative stress represents a hallmark of sleep disorders, while sleep deprivation itself can exacerbate oxidative processes. Antioxidative compounds, such as crocetin [52], ergothioneine (EGT) [55], chlorogenic acids (CGA) [58], ashwagandha [48,61], and traditional herbal supplements [43], have a positive effect in improving sleep disorders. Furthermore, Jiang et al.’s study [98] provides new insights into the mechanisms of dietary antioxidants in sleep regulation. Adequate antioxidant intake helps scavenge free radicals and reduces reactive oxygen species production, mitigates brain damage, and regulates the levels of neurotransmitters such as GABA, thereby improving sleep quality [99].

## 5. Strengths and Weaknesses

In this systematic review and meta-analysis, we assessed combining subjective sleep quality assessment indicators (PSQI scores) and objective ratings (SE, TST, SL, WASO, and NASO) of instrumental recordings, and there were significant differences between dietary supplementation and no dietary supplementation in terms of SE, TST, SL, WASO, and NASO and the mechanism of action of these substances to improve sleep quality. This may provide a comprehensive understanding of how dietary supplement interventions affect sleep health.

However, this review still has weaknesses. One of the main weaknesses is the limitation of the included studies for each food category regarding quantitative analysis. This is especially important for the analysis of high heterogeneity, the substances that play a major role, and the most potent substances. This study combined isolated supplement interventions with overall dietary interventions to obtain an overall estimate of the impact of nutritional interventions on sleep. This approach, however, may introduce conceptual and methodological heterogeneity due to differences in intervention form, compliance, and bioavailability. And the different intervention dosages will also cause high heterogeneity. More importantly, uncontrolled confounders such as gender, lifestyle factors, and baseline sleep severity will influence outcome accuracy. In further research, the factors mentioned above must be taken into consideration to settle this study’s limitations regarding the generalization of conclusions.

Finally, while our meta-analysis synthesizes available global evidence, the geographical distribution of the included studies is not uniform, with a predominance of data from America, East Asia, and Europe. This imbalance means our findings may not fully capture the distinct etiologies, cultural contexts, and resource availability affecting sleep in other regions, such as Africa or South America. The causes and manifestations of sleep deprivation are known to be influenced by local social and economic factors, occupational structures, and healthcare access. Therefore, the direct applicability of our conclusions to all continents should be interpreted with caution.

## 6. Conclusions

Dietary supplementation strategies have shown significant potential in improving sleep quality among individuals with sleep disorders. And it can improve PSQI score, improve SE and TST, and reduce SL and WASO. This approach involves targeted incorporation of specific nutrients, including the following:

- Amino acid (e.g., Tryptophan) and vitamin (vitamin D) sources.
- *n*-3 PUFAs.
- Essential minerals (e.g., zinc).
- Antioxidant-rich dietary components.

Practical implementation emphasizes increased consumption of legumes, whole grains, fatty fish, and dark green vegetables, coupled with reduced caffeine intake. Crucially, successful intervention requires coordination:

- Nutrient precision—strict monitoring of supplementation dosage.
- Lifestyle integration—synergistic dietary adjustments.

These multidimensional interventions collectively establish a biochemical foundation for ultimately optimizing therapeutic outcomes in sleep management. There are still many shortcomings in our research, and future research should investigate these modalities further.

## Figures and Tables

**Table 1:** PICOS criteria for inclusion in the study.

Parameter	Description
Population	Adults ≥ 19 years, healthy or just suffering from sleep disorders related to diseases
Intervention	Consuming dietary supplement(s) or food(s) rich in dietary nutrients that can improve sleep quality
Comparison	Not consuming dietary supplement(s) or food(s) rich in dietary nutrients with the function of improving sleep quality, placebo, or standardized diet
Outcome	Sleep quality outcomes (SE, SL, TST, WASO, NASO, and PSQI)
Study design	RCT

**Table 2:** Result of meta-regression for PSQI.

Sources of Heterogeneity	Coefficient	*p*	95% CI	
Health conditions	0.082	0.629	−0.286	0.450
Country	−0.112	0.015	−0.196	−0.028
Intervention time	−0.688	0.004	−1.096	−0.281
Intervention type	−0.191	0.002	−0.294	−0.087

**Table 3:** Result of meta-regression for SE.

Sources of Heterogeneity	Coefficient	*p*	95% CI	
Health conditions	0.0452	0.811	−0.346	0.437
Country	−0.0823	0.080	−0.176	0.011
Intervention time	−0.172	0.210	−0.449	0.106
Intervention type	−0.171	0.024	−0.317	−0.025

**Table 4:** Result of meta-regression for TST.

Sources of Heterogeneity	Coefficient	*p*	95% CI	
Health conditions	0.410	0.074	−0.045	0.866
Country	−0.159	0.005	−0.261	−0.058
Intervention time	−0.385	0.010	−0.664	−0.106
Intervention type	−0.078	0.247	−0.215	0.060

## Data Availability

Data will be made available upon reasonable request.

## Supplementary Materials

The following supporting information can be downloaded at https://www.mdpi.com/article/10.3390/nu17243952/s1: Table S1: Literature retrieval strategy of each database; Table S2: Participants and dietary supplement intervention characteristics of included studies; Table S3: GRADE assessment; PRISMA_2020_checklist-update. Ref. [100] is cited in the Supplementary Materials file.

## Abbreviations

The following abbreviations are used in this manuscript:

PSQI	Pittsburgh Sleep Quality Index
SE	Sleep efficiency
TST	Total sleep time
SL	Sleep latency
WASO	Wake after sleep onset
NASO	Number of awake after sleep onset
GABA	γ-aminobutyric acid
RCT	Randomized controlled trials
CBT-I	Cognitive behavioral therapy for insomnia
M	Mean
SD	Standard deviation
MD diff	Changes in mean
SD diff	Changes in standard deviation
SMD	Standardized mean difference
r	Correlation coefficient
CI	Confidence interval
min	Minutes
MT	Melatonin
NREM	Non-rapid eye movement
5-HT	5-hydroxytryptophan
NAT	*N*-acetyltransferase
SNAT	Serotonin *N*-acetyltransferase
ASMT	Acetylserotonin O-methyltransferase
SCN	Hypothalamic-suprachiasmatic nucleus
TPH2	Tryptophan hydroxylase 2
PUFA	Polyunsaturated fatty acid
