# Supplementary material for: Dietary Supplement Interventions and Sleep Quality Improvement: A Systematic Review and Meta-Analysis
Source: Nutrients. 2025 Dec 17;17(24):3952. doi: 10.3390/nu17243952 (PMC12736316; doi:10.3390/nu17243952)
Supplement: Supplementary file 1 [file nutrients-17-03952-s001.zip › Table S3 GRADE assessment_.pdf]

Author(s):
Question: foods or dietary supplements intervention compared to placebo for sleep disorder
Setting:
Bibliography: . foods or dietary supplements intake versus placebo for sleep disorder. Cochrane Database of Systematic Reviews [Year], Issue [Issue].

Certainty assessment							№ of patients		Effect		Certainty	Importance
№ of studies	Study design	Risk of bias	Inconsistency	Indirectness	Imprecision	Other considerations	Intervention	placebo	Relative (95% CI)	Absolute (95% CI)		
**PSQI**												
11	randomised trials	serious[a]	not serious	not serious	serious[b]	none	372	356	-	MD **0.56 lower** (1.16 lower to 0.04 higher)	⊕⊕◯◯ Low[a,b]	IMPORTANT
**Sleep efficiency**												
22	randomised trials	serious[a]	not serious	not serious	not serious	none	569	511	-	MD **2.58 higher** (2.01 higher to 3.16 higher)	⊕⊕⊕◯ Moderate[a]	CRITICAL
**Sleep latency**												
20	randomised trials	serious[a]	not serious	not serious	not serious	none	451	437	-	MD **0.6 lower** (1.01 lower to 0.18 lower)	⊕⊕⊕◯ Moderate[a]	CRITICAL
**Total sleep time**												
18	randomised trials	serious[a]	not serious	not serious	not serious	none	441	389	-	SMD **0.33 higher** (0.08 higher to 0.57 higher)	⊕⊕⊕◯ Moderate[a]	CRITICAL
**Wake after Sleep Onset Time**												
12	randomised trials	serious[a]	not serious	not serious	not serious	none	274	235	-	SMD **0.3 lower** (0.48 lower to 0.12 lower)	⊕⊕⊕◯ Moderate[a]	CRITICAL
**Number of Wake after Sleep Onset**												
6	randomised trials	serious[a]	not serious	not serious	serious[b]	none	172	147	-	MD **1.57 lower** (3.16 lower to 0.02 higher)	⊕⊕◯◯ Low[a,b]	CRITICAL

CI: confidence interval; **MD:** mean difference; **SMD:** standardised mean difference

Explanations

a. Existence of attrition and performance bias in the literature was assessed as high risk.
b. The confidence interval crosses the invalid line, and the existing evidence cannot confirm that the intervention is effective, nor can it exclude its non-significant effect, and cannot provide accurate estimates for clinical decision-making.
