# Supplementary material for: Dietary Supplement Interventions and Sleep Quality Improvement: A Systematic Review and Meta-Analysis
Source: Nutrients. 2025 Dec 17;17(24):3952. doi: 10.3390/nu17243952 (PMC12736316; doi:10.3390/nu17243952)
Supplement: Supplementary file 1 [file nutrients-17-03952-s001.zip › Table S1 in manuscript.pdf]

Table S1 Literature retrieval strategy of each database

Database	Search formula
PubMed	((food [MeSH] OR "food" OR "foods" OR (food supp*)) OR (diet [MeSH] OR "diets" OR (diet supp*))) AND (sleep [MeSH] OR "sleep" OR "sleep duration" OR "sleep efficiency" OR "sleep latency" OR "sleep quality" OR "sleep index") AND (randomized controlled rial [MeSH] OR "randomized controlled trial" OR "randomized controlled trials" OR "RCT" OR (random*)) （Filter，human and adult 19+）
Web of Science	(TS=(sleep) OR "sleep" OR "sleep quality" OR "sleep disorders" OR "sleep deprivation" OR "sleep duration") AND (TS=(food) OR "diet" OR "food" OR "food intake" OR "nutrition" OR "nutrient") AND (KP=(human) OR "adult") AND (KP=(RCT) OR "randomized controlled trial" OR "randomized controlled trials" OR "RCT")
CNKI	Theme : (food + food source + nutritional food + food intake + different food + resident food) 0R (Theme : diet + dietary intake + dietary nutrients + dietary nutrition + dietary guidance) OR (Theme : nutrition + nutrients + nutritional status + nutrients + nutrients + nutrients + nutritional support) OR (Theme : diet + reasonable diet + diet therapy + diet intervention + diet adjustment + diet therapy) AND (Summary : improving sleep + improving sleep quality + improving sleep status +

	improving sleep function + improving sleep function + improving sleep health food (accurate))
Science Direct	Title, abstract, keywords: "RCT" OR "randomized controlled trial" OR "randomized controlled trials"<br>Title: ("sleep" OR "sleep quality" OR "sleep duration") AND ("food" OR "foods" OR "food intake" OR "diet" OR nutrition OR nutrient))
Wiley	"(food [MeSH] OR "food" OR "foods" OR (food supp*) OR "food intake") OR (diet [MeSH] OR "diets" OR (diet supp*)) OR ("nutrition" OR "nutrient")" in Title and ""sleep" OR "sleep quality" OR "sleep duration" OR "sleep disorder"" in Abstract
CVIP	Title = food + food source + nutritional food + food intake + different food + resident food OR Title = diet + dietary intake + dietary nutrients + dietary nutrition + dietary guidance OR Title = diet + reasonable diet + diet therapy + diet intervention + diet adjustment + diet therapy OR Title = nutrition + nutrients + nutritional status + nutrients + nutrients + nutritional support AND Abstract = : improve sleep + improve sleep quality + improve sleep status + improve sleep + improve sleep function + improve sleep function + improve sleep health food
