# Supplementary material for: Dietary Supplement Interventions and Sleep Quality Improvement: A Systematic Review and Meta-Analysis
Source: Nutrients. 2025 Dec 17;17(24):3952. doi: 10.3390/nu17243952 (PMC12736316; doi:10.3390/nu17243952)
Supplement: Supplementary file 1 [file nutrients-17-03952-s001.zip › Table S2 characteristics_revision.pdf]

Table S2 Participants and dietary intervention characteristics of included studies

Author	Participant	Country	Sleep quality measurement	Gender	Intervention duration	Intervention used	Dosage
Baba, Y. et.al. (2024) [37]	Healthy people	Japan	PSQI, SE, TST, SL, WASO, NASO	Man and woman	4 weeks	Matcha capsules	2700 mg matcha
Baradari, Afshin Gholipour et.al. (2018) [38]	ICU nurse	Iran	SL	Man and woman	A month	capsule of Zn sulfate	220 mg Zn sulfate
García-García Carolina et.al.	no chronic disease history	South Korea	PSQI,	Not reported	A month	Group A γ-PGA supplementation,	group A γ-PGA 600 mg, group B VitB6

(2021) [39]	adult					Group B VitB6 supplementation, Group C a combined γ-PGA and VitB6 supplementation.	100 mg, and group C both γ-PGA 600 mg and VitB6 100 mg
Cornu Catherine et.al. (2010) [40]	Adult with a chronic primary insomnia	France	SE	Man and woman	4 weeks	Capsules contained the dietary supplement under the brand name Cyclamax® and Soya oil (Glycine max).	Cyclamax® contains 260 mg and Soya oil 173 mg.

Yang Cong et.al. (2024) [41]	people with PSQI scores ≥ 6	China	PSQI, TST	Not reported	2 weeks	Group A: products with active ingredients GABA and asparagus powder; group B: products with active ingredients GABA	Group A: GABA 120mg and asparagus powder 1500mg; group B: GABA 120mg
Drennan, M. D. et.al. (1991) [42]	Healthy paid volunteer	America	SE, TST, SL, WASO	Male	2 weeks	oral microencapsulated potassium chloride supplements	96-mEq/day potassium chloride
Farag, N. H.	healthy	America	SL	Not	4 nights	traditional herbal	160 mg/tablet

et.al. (2003) [43]	volunteers suffering from sleep onset insomnia			reported		supplement	
Garrido, M. et.al. (2013) [44]	Healthy adult	Spain	SE, TST, SL, NASO	Man and woman	1 week	A Jerte Valley cherry-based product (JVCP)	27.85 g/each dosage
Ha, E. et.al. (2019) [45]	Adul had mild insomnia	South Korea	SE, TST, WASO	Man and woman	4 weeks	Polygonatum sibiricum (PS) rhizome extract (n = 40)	500 mg/day
Hansen, A. L. et.al. (2014)	21-60 years Adult	America	SE, TST, SL, WASO	Man	5 months	Atlantic salmon	150 g

[46]							
Hayat, Mariya et.al. (2023) [47]	35.4 ± 8.9 years healthy adult	America	PSQI, SE, TST, SL, WASO, NASO	Man and woman	16 weeks	group: 1) HPWP- High protein diet (30% protein) with white potatoes; group 2) HPCC- High protein diet (30% protein) with a control carbohydrate (white rice)	potato and rice was equal to 1/2 cup and 1/3 cup respectively.
Saito Hitomi et.al. (2017)	Healthy adult	Japan	PSQI, SE, TST, SL	Man and	12 weeks	Group A zinc food; Group B zinc plus	150 mg/day containing 10% zinc,

[48]				woman		astaxanthin food	group B 3.3 mg/day astaxanthin extra
Howatson, G. et.al. (2012) [49]	Healthy adult	Britain	SE, TST, SL,	Man and woman	7 days	Tart cherry juice concentrate	30 mL
Hudson, C. et.al. (2010) [50]	Healthy adult	Cannada	SE, TST, WASO	Man and woman	3 weeks	Food1 contained deoiled butternut squash seed meal and dextrose. Food 2 contained pharmaceutical tryptophan, dextrose and rolled	food 1 25 mgdeoiled butternut squash seed meal and of dextrose,respectively; Food 2 250 mg pharmaceutical tryptophan, 25 g of dextrose and 25 g of

						oats.	rolled oats
Priya Khare et.al. (2020) [51]	Healthy adult	India	PSQI	Woman	90 days	NRL/2019/5PNW protein blend supplements	dose of 10 gm. in the morning with 150ml of milk
Kuratsune, H. et.al. (2010) [52]	PSQI scores ≥ 6	Japan	SE, SL	Man	2 weeks	Crocetin supplement	7.5 mg per capsule
Su Eun Lim et.al. (2024) [53]	Adult experienced sleep disturbances	South Korea	SE, TST, SL, WASO	Man and woman	8 weeks	LTC-022 (The main ingredients are Lactium and L-theanine)	Lactium 500 mg/tablet (white tablet, containing 60% Lactium, 15% crystalline cellulose, 12.5% dextrin, and

							other ingredients), L-theanine 700 mg /tablet (grey tablet, containing 29.16% L-theanine, 30% maltodextrin, 19.789% crystalline cellulose, and other ingredients)
Majid, M. S. et.al. (2018) [54]	Adult with sleep disorder	Iran	PSQI, SE, SL	Man and woman	8 weeks	Vitamin D supplement	four edible pearls, each 50 000 IU vitamin D
Katsube	adult with	Japan	PSQI,	Man	4 weeks	Orally ingested	20 mg

Makoto et.al. (2022) [55]	reported high anxiety and sleep complaints			and woman		ergothioneine (EGT)	
Yang TH et.al. (2020) [56]	postmenopausal women	China	PSQI, SE, TST, SL	Woman	8 weeks	Tomato	250 g
Oberther, Tiffany J. et.al. (2024) [57]	Full-time firefighter	America	SE, TST, SL, WASO, NASO	Not reported	8 weeks	Peanut butter	32 g
Park, I. et.al. (2017) [58]	Healthy adult	Japan	SE, TST, SL	Man and	5 days	Beverage of Chlorogenic acids	600 mg CGA

				woman		(CGA)	
Patan, M. J. et.al. (2021) [59]	Healthy adult	Britain	SE, TST, SL, WASO, NASO	Man and woman	26 weeks	Group A DHA-rich capsules ; Group B EPA-rich capsules	Group A: 900 mg DHA/d and 270 mg EPA/d); Group B: 360 mg DHA/d and 900 mg EPA/d
Pigeon, W. R. et.al. (2010) [60]	Oder adult with insomnia but otherwise healthy	America	SE, TST, SL, WASO	Not reported	2 weeks	Tart cherry juice beverage	drink two 8-ounce servings of the assigned beverage
Pérez Piñero，Silvia et.al. (2024) [61]	healthy adults with sleep problems	Spain	SE, SL	Man and woman	90 days	Group A ashwagandha, Group B	Group A: 250 mg; Group B: 250 mg ashwagandha plus

						ashwagandha plus tryptophan , Group C ashwagandha	175 mg tryptophan; Group C: 600 amgashwagandha
Silvia Pérez Piñero et.al. (2024) [62]	Healthy individuals with sleep problems	Spain	PSQI, SE, TST, SL, WASO, NASO	Man and woman	90 days	An extract of Aloysia citrodora (lemon verbena)	
Um, M. Y. et.al. (2018) [63]	Adult self-who reported sleep disturbances	South Korea	PSQI, SE, TST, SL, WASO	Man and woman	1 week	Phlorotannin supplement	500 mg/day
Um, M. Y. et.al. (2019) [64]	Adult with sleep disorders	South Korea	PSQI, SE, TST, SL, WASO	Man and woman	2 weeks	rice bran extract supplement	1,000 mg/day
